# Two Markov Solution Process Models for the Assessment of Planning in Problem Solving

**DOI:** 10.1017/psy.2025.10042

**Published:** 2025-11-13

**Authors:** Andrea Brancaccio, Debora de Chiusole, Ottavia M. Epifania, Pasquale Anselmi, Matilde Spinoso, Noemi Mazzoni, Alice Bacherini, Matteo Orsoni, Sara Giovagnoli, Irene Pierluigi, Mariagrazia Benassi, Giulia Balboni, Luca Stefanutti

**Affiliations:** 1 Department FISPPA, University of Padua, Italy; 2 Department of Psychology “Renzo Canestrari,” University of Bologna, Italy; 3 Department of Philosophy, Social Sciences and Education, University of Perugia, Italy

**Keywords:** Markov models, problem space, problem-solving modeling, procedural knowledge space theory, tower of London test

## Abstract

Tower tasks are popular tools used to measure planning skills. The sequences of moves undertaken by the respondents in solving tower tasks might provide important and useful information to shed light on their planning skills. The article focuses on the distinction between a situation where planning occurs before action (pre-planning) from one where planning and action are interlaced all along the execution of the task (interim-planning). While the model for pre-planning was already developed by Stefanutti et al. (2021), an alternative model for the interim-planning is proposed. The two models are compared with one another in an empirical study. In accordance with the literature on the development of planning skills, the pre-planning model better fits data collected on individuals aged 14 on, while the interim-planning model displays a better fit with data collected on individuals aged 4–8. This result is further corroborated by the analysis of the time performance.

## 1 Introduction

Planning is the ability to identify and organize the sequence of actions necessary to carry out goal-directed behaviors (Cristofori et al., 2019; Lezak et al., 2004). In clinical and experimental neuropsychology, tower tasks, such as the tower of London test (ToL test; Shallice, 1982) and its variants (Berg & Byrd, 2002; Unterrainer et al., 2005) are popular tools used to measure this ability (Anderson & Reidy, 2012; McCormack & Atance, 2011; Sullivan et al., 2009). These tasks require planning in terms of means-ends analysis to solve a series of increasingly challenging sequential problems (Krikorian et al., 1994).

Despite its popularity, fundamental questions about what exactly the ToL measures remain unsolved (Georgiou et al., 2017). For example, Leontjev (1978) proposed three levels of planning (i.e., activity, action, and operations) but it is unclear which one the ToL assesses. Some argue it captures high-level cognitive control (e.g., Morris et al., 1993; Shallice, 1982), while others suggest it reflects lower-level procedural planning (e.g., Ward & Allport, 1997) or working memory demands (e.g., Phillips et al., 1999). Additionally, different scoring methods (e.g., first move time, total correct, etc.) may not measure the same construct, complicating interpretation. Indeed, a large corpus of literature analyzed the performance at tower tasks considering the accuracy of the performance (i.e., the number of problems correctly solved, either at the first attempt or with multiple attempts), while fewer studies have focused on the time performance (Asato et al., 2006; Luna et al., 2004), distinguishing between the time spent in pre-planning the solution of the task (i.e., the time elapsed between the presentation of the problem and the first move) and the execution time (i.e., the time elapsed between the first move and the completion of the task). This lack of clarity affects clinical and developmental research, highlighting the need for studies that distinguish planning from other executive functions.

A road for clarifying the relationship between ToL performance and distinct levels of planning could be derived from the integration of process-tracing methods, such as eye-tracking, response-time modeling, or model-based approaches that could provide deeper insights into planning processes. For example, Mitsopoulos et al. (2015) applied reinforcement learning models to ToL performance, highlighting how different problem-solving strategies emerge over time. Similarly, Köstering et al. (2012) used a latent-class approach to identify distinct patterns of planning impairment in clinical populations. These studies suggested the need for a more nuanced understanding of planning, moving beyond simple accuracy-based measures to computational and latent-variable analyses that capture the dynamics of decision-making and strategy selection.

Similarly to what is proposed here, these works have the novelty of considering the so-called “problem space” of the ToL (e.g., Berg & Byrd, 2002; Stefanutti, 2019). The problem space refers to the complete set of possible states, transitions, and solution paths that define the solution process of the problems. Using the formal modeling of the problem space in the ToL task might offer significant advantages in assessing individuals’ planning abilities. Indeed, unlike traditional scoring methods that focus on the number of moves or rule violations, a problem-space-based model allows for a deeper understanding of an individual’s planning abilities. Indeed, the solving of the ToL test problems involves the planning of *sequences of moves*. Recording the entire solution process might be difficult when administering the physical version of the test, but not when a computerized version is used. The analysis of the sequences of moves might provide insights into the cognitive processes involved in the resolution of tower tasks. To the best of our knowledge, only two studies considered the specific sequences of moves. Specifically, Stefanutti et al. (2021) proposed a mathematical framework based on procedural knowledge space theory (PKST) to model the solution process behind tower tasks. A critical assumption of this model, named pre-planning assumption, is coherent with the idea that the problem solver plans the entire sequence of moves in advance. Although this assumption is plausible in certain populations (e.g., young adults), it might not be in others (e.g., children or clinical populations). In this regard, Brancaccio et al. (2023) proposed a model based on an alternative assumption, named interim-planning, which is coherent with the idea that the planning process might be fragmented throughout the execution of the problem. While a model based on the pre-planning assumption has already been empirically validated, a model based on the interim-planning assumption has not.

The aims of this study are to (i) provide the algorithms for the maximum likelihood (ML) parameter estimation of the interim-planning model, (ii) empirically validate it, (iii) compare the pre-planning and interim-planning models in populations of different ages, and (iv) externally validate these models considering the time performance of the respondents.

The manuscript is organized as follows: Section 2 provides a brief literature review on the measurement of planning skills through tower tasks (Section 2.1) as well as an introduction to knowledge space theory (KST) (Section 2.2) and PKST (Section 2.3). Section 3 thoroughly presents the Markov solution process models (MSPMs), along with the two assumptions consistent with the pre-planning and the interim-planning strategies. Section 4 illustrates a simulation study carried out for investigating the parameter recovery of the model based on the interim-planning assumption. Then, Section 5 presents the results of an empirical study where the data of a tower task are analyzed with the two models, along with the analysis of the response times. Finally, Section 6 concludes the argumentation.

## 2 Backgrounds

### 2.1 Assessment of planning with the tower of London test

The original version of the ToL test (Shallice, 1982) consists of three differently colored beads placed on three vertical pegs of different heights that may hold at maximum either one, two, or three beads, respectively. The task requires to transform the start state into a goal state with a sequence of actions, each of which consists of moving one bead from one peg to another, in accordance with specific rules and given the number of moves. A problem is correctly solved when the goal state is achieved within the minimum number of moves.

During the execution of the ToL task, many indices can be recorded—such as accuracy, solution time, number of moves, sequences of moves, and rule violations. The most frequently used behavioral measures are accuracy, pre-planning time, and execution time (e.g., Anderson et al., 1996; Culbertson & Zillmer, 1998; Krikorian et al., 1994; Unterrainer et al., 2003).

Evidence from developmental studies using accuracy as a performance indicator shows a gradual growth in planning skills from childhood to adolescence and early adulthood (Albert & Steinberg, 2011; Asato et al., 2006; Crone & Steinbeis, 2017; De Luca et al., 2003; Korkman et al., 2001; Luciana & Nelson, 1998; Luciana et al., 2009). Specifically, traditional and computerized versions of ToL test indicate that planning ability improves with age, reaching adult-like levels by 14 years (De Luca et al., 2003; Krikorian et al., 1994; Luciana & Nelson, 1998, 2002; Malloy-Diniz et al., 2008). This trend is consistent with findings using the Tower of Hanoi, which show progressive planning improvement during childhood (Díaz et al., 2012).

Findings on age-related changes in planning time are mixed. Some studies report increased planning time with age, particularly in complex tasks (Asato et al., 2006; Filippi et al., 2020; Steinberg et al., 2008), while others show a decrease during childhood and early adolescence (Huizinga et al., 2006; Injoque-Ricle et al., 2014). Task difficulty appears to modulate these effects across age groups.

The aforementioned studies used pre-planning time as an index of planning skills. However, it is conceivable that, in some cases, planning during the ToL task does not end when the first move is made, but extends throughout the task. Indeed, the execution phase likely encompasses not only the motor actions of bead movement, but also the cognitive processes involved in ongoing online planning and error correction (Berg & Byrd, 2002; Luciana et al., 2009). Notably, to our knowledge, there is a paucity of research that attempts to isolate, differentiate, or quantify, within the execution phase, the time spent on movement and the time spent thinking. Indeed, there have been attempts to reduce the planning before the first move (Owen et al., 1995; Phillips et al., 2001; Ward & Allport, 1997); however, no study has measured planning time within the execution phase. In the traditional paper-and-pencil versions of the ToL, the distinction between movement and planning time is not typically provided, and would pose a challenge for the experimenter to measure it. Nevertheless, computerized versions offer the possibility to dissect these processes easier, potentially revealing valuable information and insights into the diverse strategies individuals employ to complete the task.

### 2.2 Knowledge space theory

KST (Doignon & Falmagne, 1985, Doignon & Falmagne, 1999, Falmagne & Doignon, 2011) is a mathematical framework developed for the efficient and accurate assessment of knowledge. At its core is the concept of a *knowledge state*, denoted as a set 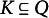 , representing the problems that an individual masters within a specific collection of problems *Q* named the *knowledge domain*. The entire collection of possible knowledge states for a population is a *knowledge structure* 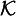 . When a knowledge structure is closed under union^1^ it is called a *knowledge space*.

Various methods have been developed over the years to construct a knowledge structure. These methods include exploiting the relation between the problems (e.g., Dowling, 1993; Koppen, 1993; Stefanutti & Koppen, 2003), assuming a competence level along with a set of underlying skills (e.g., Heller, Augustin, et al., 2013; Heller, Ünlü, et al., 2013; Korossy, 1999), employing data-driven procedures (e.g., de Chiusole et al., 2017, 2020; Spoto et al., 2016), or deriving a knowledge space from a problem space underlying the tasks (Stefanutti, 2019). This last approach is the one adopted in this article.

The most common probabilistic model in KST is the basic local independence model (BLIM; Falmagne & Doignon, 1988). The BLIM is a restricted latent class model where the latent classes represent the knowledge states. In this model, what is observed is a response pattern, denoted as 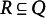 , which indicates the subset of items correctly solved by each individual. Given a population of individuals, a probability distribution 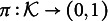 on the knowledge states is assumed. Moreover, two parameters estimated for each item 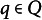 are considered: the conditional probability of observing a wrong answer for item *q* given that 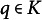 , denoted 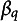 ; the conditional probability of observing a correct answer for item *q* given that 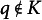 , denoted 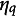 . Both parameters have a typical interpretation in the BLIM: 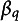 is a careless error probability for the item *q* and 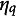 is a lucky guess probability for the same item.

In the BLIM, the restriction 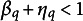 for each item is a required property for obtaining a consistent assessment of the knowledge state, that is, for identifying the knowledge state of an individual based on the observed item responses of this individual. Specifically, the probability of making a careless error on item *q* should be lower than the probability of failing *q* due to lack of mastery. On the other hand, the probability of correctly solving an item because it is mastered must exceed that of guessing it. The above restriction is called the “monotonicity condition.”

The 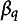 , 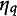 , and 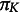 parameters of the BLIM can be estimated by ML via the expectation–maximization algorithm (Stefanutti & Robusto, 2009), by minimum discrepancy (MD, Heller & Wickelmaier, 2013) or by an estimation procedure that is a mix of the two (MDML, Heller & Wickelmaier, 2013). Methods are also available for computing ML estimates of the model parameters in the presence of missing data (Anselmi et al., 2016; de Chiusole et al., 2015). Once the BLIM based on a specific knowledge structure 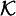 has been estimated and validated on an appropriate sample of individuals, the model parameter estimates can be used to identify the knowledge state 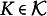 of each individual based on their item responses. This allows for the assessment of individual differences. Since, in general, knowledge states are partially ordered, the assessment is multidimensional.

### 2.3 Procedural knowledge space theory

The notion of *problem space* introduced by Newell & Simon (1972) is a commonly used concept in the study of human problem solving. The connection between KST and the problem space theory of Newell & Simon (1972) was first pointed out by Stefanutti & Albert (2003). The theoretical foundations were established by Stefanutti (2019), and take the name of PKST. The remainder of the section delves into the fundamental concepts of PKST.

Let 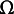 be a set of operations. A string of length *n* of elements in 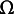 is a sequence 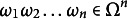 . The *concatenation* between two strings 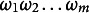 and 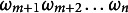 is the string 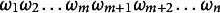 . The collection of all the strings of operations of finite length is 

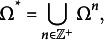

where 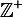 is the set of non-negative integer numbers. The empty sequence 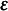 belongs to 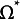 (i.e., 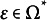 ).

In PKST, a *problem space* is defined as a triple 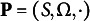 , where *S* is a nonempty set of *problem states*, 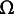 is a nonempty set of *operations*, and 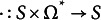 is an operator satisfying, for all 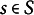 and 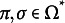 , the two axioms:

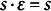
 ;

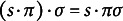
 .

The operator  is named *operation application*. Problem states and operations are primitive concepts in PKST.

A *problem* within the problem space 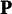 is a pair 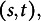 where 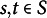 and there exists a string 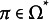 such that 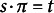 . The collection of all the problems in 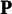 is thus 

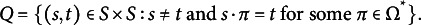

Notably, the set *Q* corresponds to the domain of knowledge in KST. In the problem space 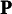 , a solution path is any pair 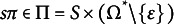 . In addition, a solution path 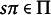 is said to *solve* the problem 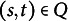 if 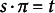 . It is worth mentioning that the same problem may be solved by multiple solution paths. A solution path 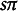 is a *subpath* of another solution path 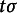 (denoted by 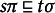 ) if there are 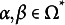 such that 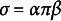 and 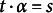 . The relation 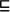 , called *sub-path relation,* is a partial order for the set 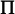 . A subset 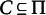 is said to *respect path inclusion* if the condition 

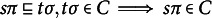

is respected for all 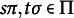 . A subset of solution paths respecting path inclusion is named a *competence state* of the problem space 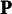 . The collection 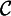 of all the competence states is the *competence space*.

The function 
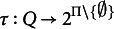
 maps each problem 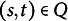 to the collection 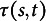 of all solution paths that solve it.

The collection of all the problems in *Q* that can be solved by an individual whose competence state is 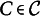 is 

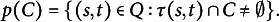

This collection 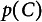 is named the *knowledge state* delineated by competence state *C*. It is evident that a problem 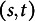 belongs to 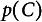 if and only if *C* includes at least one solution path solving 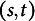 . The collection 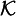 of all the knowledge states is the knowledge space derived from the problem space 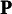 . Stefanutti (2019) introduced an algorithm for the automatic derivation of a knowledge space from a problem space. Like KST, PKST also classifies individuals based on their knowledge states, allowing for the assessment of individual differences in problem-solving skills.

A particular kind of problem space called “goal space” has been introduced in Stefanutti et al. (2021) and it is central in this work. In a goal space, each step of the solution process of a problem is classified as “correct-so-far” or “incorrect.” A goal space is defined as a problem space 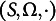 with two distinct states 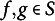 such that:

1. for all 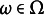 , 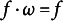 and 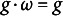 ;
2. for each 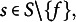 there is a string 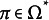 such that 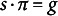 .

The problem states *f* and *g* are called the *failure* and the *goal* states, respectively, and a goal space is denoted by the 5-tuple 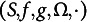 . A solution process is deemed “correct” when it reaches *g* and “incorrect” when it reaches *f*.

According to property (GS1), both the failure and the goal states are considered final states, meaning that any operation applied to either of them is ineffective. Property (GS2) stipulates that the goal state *g* is reachable from any problem state except *f*. Consequently, any pair 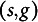 with 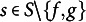 constitutes a problem within the goal space.

### 2.4 An example with the tower of London

**Figure 1:**
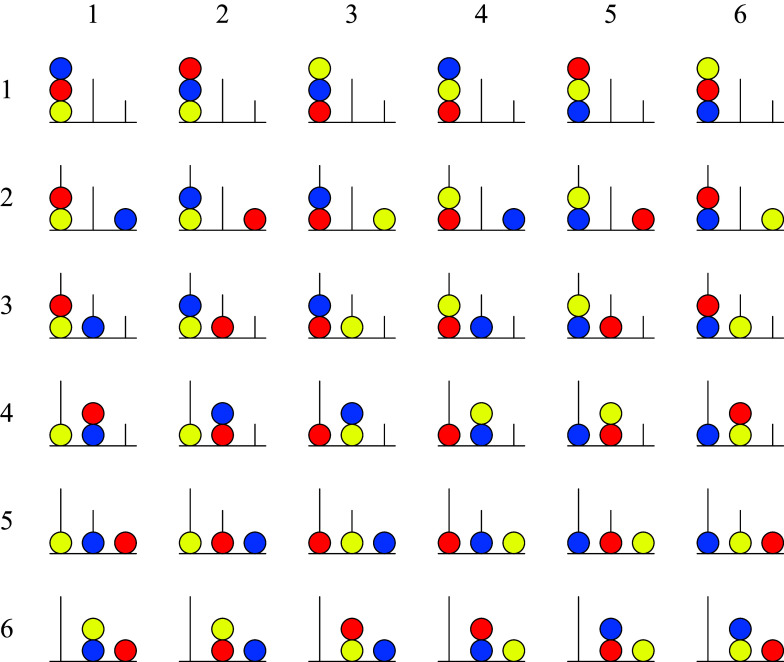
The 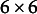 different problem states of the ToL test.

In this section, an example based on the problem space of the ToL test is presented to provide a practical illustration of the theoretical concepts introduced in the previous section. In the ToL test, a problem state consists of three beads of different colors placed on three pegs of different heights mounted on a wooden support. The three beads can be moved, one by one, from one peg to another. The number of possible problem states in the ToL test problem space is 36, and they coincide with the configurations of the beads in the pegs. Figure 1 depicts the 36 problem states of the ToL. Each of them is labeled by a pair 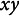 of numbers, where *x* stands for one of the six spatial arrangements (rows in the figure), whereas *y* stands for one of the color permutations (columns in the figure). An operation consists of moving a ball from one of the three pegs to another. Naming the three pegs as left, center, and right, six operations are entailed: left to center 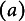 , center to left 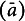 , center to right 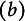 , right to center 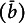 , left to right 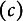 , and right to left 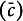 . Therefore, in the ToL, the set of operations is 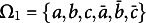 .

**Figure 2:**
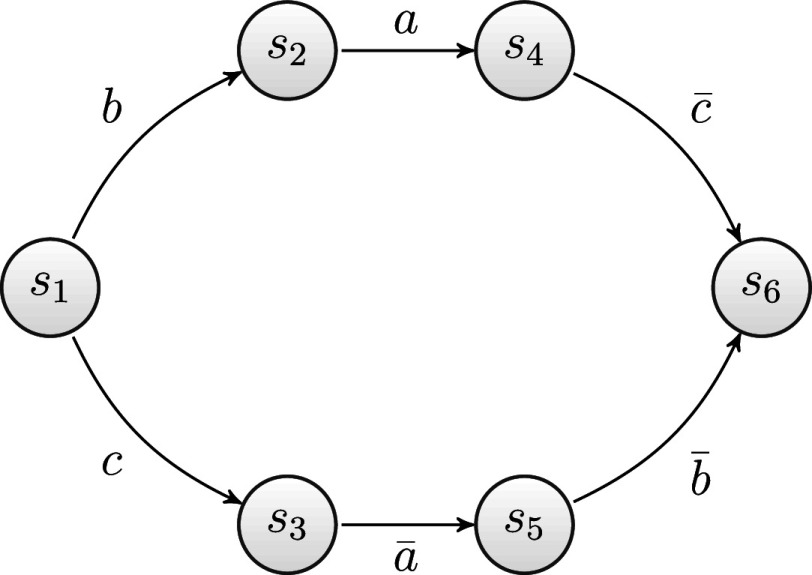
Directed graph of a portion of the ToL test problem space.

Figure 2 depicts the directed graph of a portion of the ToL test problem space in which the collection of problem states is 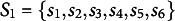 . Each problem state corresponds to a label based on the coding used in Figure 1. Specifically, 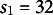 , 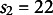 , 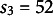 , 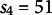 , 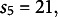 and 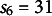 . The triple 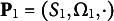 is the problem space for this portion of the ToL test problem space. We can trivially derive a goal space 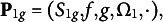 where 
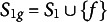
 and 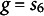 . In addition, all problem states different from the goal are connected to the failure state *f* by some operations in 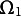 .

In the example, only the subset of problems from goal space 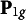 which have 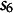 as goal state is considered, namely, 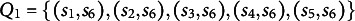 . Since all the problems in 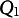 have form 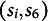 , for lightening the notation, each of them is just represented by the initial state 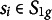 . To solve a problem, one needs to know at least one of the solution paths of that problem. For instance, problem 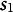 has two possible solution paths, namely, 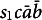 and 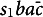 . The set of all solution paths that solves anyone of the problems in 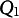 is 

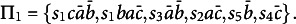

The mapping 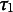 for the subset 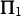 is constructed instead of deriving the mapping 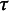 for the whole set 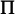 of solution paths. Thus, the mapping 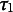 is defined as follows: 

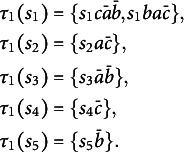



The collection of solution paths that respects path inclusion in 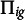 is shown in column 1 of Table 1.

**Table 1:** The 16 competence states obtained from the goal space 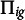 and the knowledge states delineated from them are presented in the first and second columns, respectively

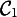	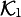
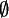	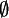
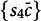	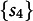
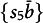	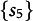
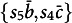	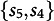
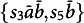	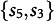
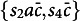	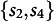
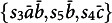	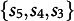
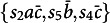	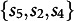
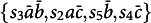	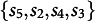
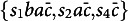	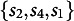
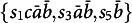	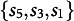
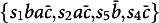	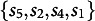
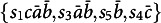	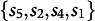
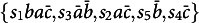	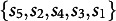
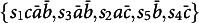	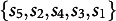
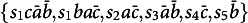	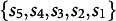

Let us consider the competence states 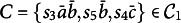 , since 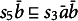 and both 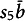 , and 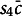 do not have any subpath indeed *C* respects path inclusion. The knowledge state delineated by *C* is obtained by a simple application of the problem function: 

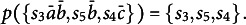

On the whole, applying the problem function *p* to each of the competence states yields the knowledge states in column 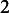 of Table 1. It is worth noting that certain knowledge states, specifically 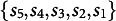 , are repeated in the table. This repetition arises because the problem function *p* is not injective.

It is worth recalling that each problem in this example is expressed only by its initial state. In general, problems in the knowledge state would be expressed as ordered pairs of initial and final states. In this example, since 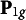 is a goal space, every knowledge state can be obtained from some competence state by just dropping the sequence of operations and retaining the initial state for each of the solution paths in the competence state.

## 3 Markov solution process models

A sequence 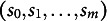 of moves that, in a goal space, leads from the initial state 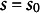 to the goal state 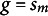 of a problem 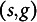 is named a *problem solution process*. Therefore, it is assumed that all the states of a problem space that are traversed by a problem solver are observable. It follows that what is observed for every single problem is not only the correctness of the problem solution, but the entire solution process.

Stefanutti et al. (2021) developed a model, named the MSPM, where the problem solution process is regarded as a random process 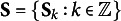 in which every 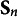 is a random variable whose realizations are problem states in *S*. The MSPM postulates a dependence of the problem solution process on the latent knowledge state of the problem solver, and assumes that, given the state of this last, the solution process is Markovian. More precisely, if *Q* is the set of all the problems having state *g* as the goal, 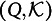 is a knowledge structure, 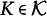 is the problem solver’s knowledge state, and 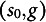 is the problem to be solved, then the probability of observing problem state 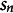 at the *n*-th step of the solution process, given the previously traversed states, and the knowledge state *K*, satisfies the Markov property 
(1)

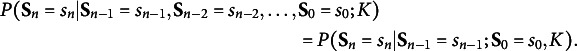

In the sequel, the probability 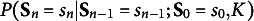 is referred to as a transition probability and, to lighten the notation, it is denoted 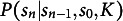 . It immediately follows from (1) that the conditional probability of an entire solution process 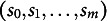 , given the knowledge state 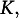 can be factored as 

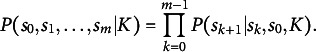



Indicating with 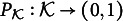 the probability distribution on the knowledge states in 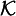 , the conditional marginal probability of observing 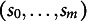 as a solution process for problem 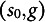 , is given by 

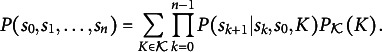



This last is the general equation of the MSPM. With no further assumptions, the number of transition probability parameters 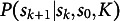 in the model may be huge, but this number can be drastically reduced. In the first place, as a necessary assumption, a transition from a state 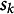 to another state 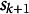 of the goal space can only occur (with a positive probability) if there is an operation 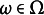 that transforms 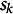 into 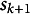 . Formally, call a *directed edge of the goal space* any ordered pair 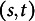 of problem states in *S* for which there is a single operation 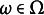 transforming *s* into *t* (i.e., such that 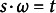 ). Then, 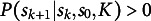 only if 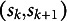 is a directed edge. Because of this constraint, many transition probabilities will be zero.

Moreover, empirically sound assumptions that allow for further reducing the total number of distinct transition probabilities that need to be estimated can be introduced. Two of these assumptions are investigated in this article. In what follows, these assumptions are labeled as (A1) and (A2). The former one was proposed and empirically tested by Stefanutti et al. (2021), whereas the latter one was proposed by Brancaccio et al. (2023), but it was never tested in empirical studies.

According to (A1) assumption, the transition probability from problem state *i* to problem state *j* is assumed to depend on the knowledge state *K* of the problem solver through the following simple mechanism:

(A1) Given any problem 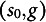 , any directed edge 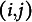 , and any knowledge state 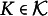 , 

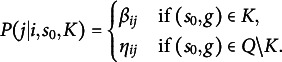

where 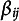 and 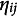 are real parameters of the single directed edge 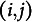 .

As such, the transition probability from *i* to *j* depends both on the knowledge state *K* of the problem solver and on the initial state 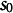 of the problem.

The alternative assumption (A2) establishes that a transition from the current state *i* to an adjacent state *j* still depends on the knowledge state *K*, but it is independent of the initial problem state 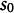 . Precisely:

(A2) For every problem 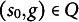 , every directed edge 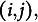 and every knowledge state 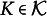 , the probability of a transition from *i* to *j* is 

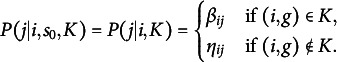

An observation concerning the two parameter types 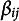 and 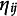 , which holds under both assumptions (A1) and (A2), is as follows. If *i* is any problem state different from both *g* and *f*, and 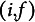 is a directed edge, then the parameter 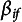 is the probability to observe a transition to the failure state: A kind of careless error. Conversely, if 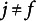 , and 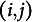 is a directed edge, then 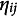 is the probability of a correct transition toward the goal: A kind of guessing.

Table 2 presents a classification of possible interpretations for all transition parameters based on Assumption (A1).

**Table 2:** Interpretation of 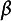 and 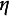 parameters on the basis of problem mastery and observed transition under (A1) assumption

Observed transition	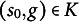 : Mastered	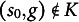 : Not mastered
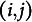 : Correct	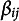 : Non-careless error	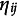 : Lucky guess
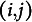 : Incorrect	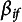 : Careless error	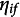 : Non-lucky guess

Table 2 refers to (A1) assumption, where the parameters’ interpretation is based on the relationship between the observed transition 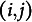 and the individual’s mastery of the initial problem 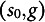 . Columns represent whether problem 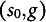 is mastered ( 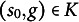 ) or not ( 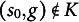 ), while rows represent the observed transition during a single step of the solution paths, which can be either correct 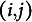 or incorrect 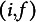 .

As the table shows, a correct transition performed by an individual who masters 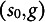 is classified as a *non-careless error*. Hence, 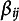 is the probability of a non-careless error. Conversely, a correct transition performed by an individual who does not master the problem is classified as a *lucky guess*. Hence, 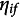 is the probability of a lucky guess, implying that the correct response occurred by chance.

An incorrect transition 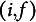 made by an individual who masters the problem 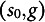 is considered a *careless error*. Hence, 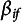 is the probability of a careless error. Finally, an incorrect transition made by an individual who does not master the problem is classified as a *non-lucky guess*. Hence, 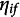 is the probability of a non-lucky guess.

In Assumption (A2), mastery is assumed for the problem at the beginning of the observed transition, 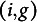 , rather than for the problem on which the entire solution process started, 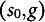 . Therefore, under (A2), the 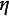 and 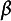 parameters receive an analogous interpretation, which is easily obtainable by replacing 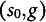 with 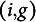 in the heading of Table 2.

A monotonicity condition similar to the one introduced in Section 2.2 is required in the MSP models: If 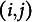 is a directed edge with 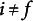 , then 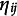 is regarded as a guessing, whereas 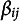 is regarded as a non-careless error, and the inequality 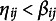 is expected. The monotonicity condition can be formally stated as follows: given any two problem states 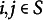 , 
(2)

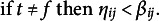

In general, under both assumptions, the transition probability of a directed edge 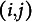 is either a 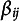 or an 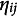 . The only difference between assumptions (A1) and (A2) is that in the latter one, this transition probability depends on the current state *i* and not on the initial state 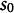 . Stated differently, while in (A1) assumption, the transition probabilities of the solution process are *globally determined* by the overall problem 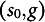 that has to be solved, in (A2) assumption, they are *locally determined* by the particular sub-problem 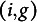 that still needs to be solved in order to reach the goal. This distinction between the two processes has several implications, leading to different predictions in the models that are based on one assumption or the other.

**Figure 3:**
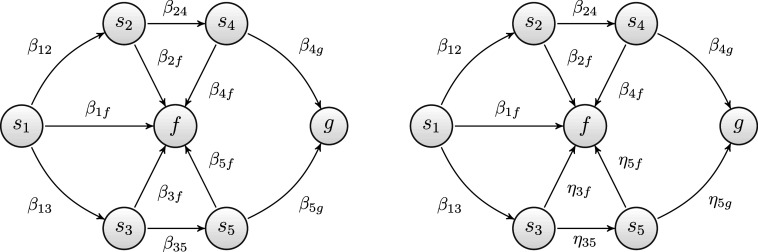
Goal spaces of Example 1, where problem 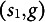 has two 3-move solution paths. *Note*: On the left side, the edges refer to transitions in the MSPM1 (pre-planning assumption), whereas on the right side, they refer to transitions in the MSPM2 (interim-planning assumption).

As an example, the two diagrams displayed in Figure 3 show two directed graphs of the same goal space, with a set of states 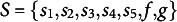 . In each graph, labeled circles represent the states of the problem space, whereas labeled edges represent moves, labeled with the names of the transition probability parameters of the MSPM. The scenario depicted in the figure reflects the case in which the problem solver’s knowledge state is 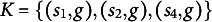 , and the problem to be solved is 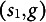 . The left-hand side diagram is based on Assumption (A1), whereas the right-hand side diagram is based on Assumption (A2). Since the problem to be solved belongs to the knowledge state *K* of the problem solver, according to (A1) (left-hand diagram), all of the transition probabilities are of type 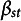 . This is not the case for (A2) (right-hand diagram). For instance, while the transition from 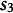 to 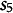 has probability 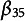 for (A1), it has probability 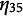 for (A2). This happens because problem 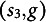 is not in *K*. Because of these differences, the same solution path may have different probabilities in the two models. For instance, the solution path passing through problem states 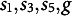 has probability 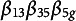 according to (A1), whereas its probability is 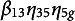 according to (A2).

**Figure 4:**
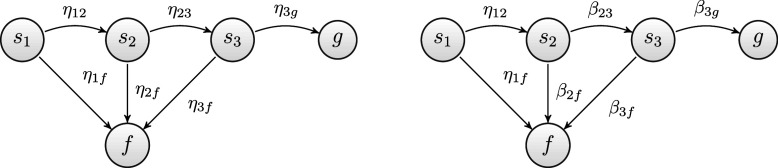
Goal spaces of Example 2, where problem 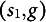 has a single 3-move solution paths. *Note*: On the left side, the edges refer to transitions in the MSPM1 (pre-planning assumption), whereas on the right side, they refer to transitions in the MSPM2 (interim-planning assumption).

Another example, similar to the previous one, is based on the two diagrams displayed in Figure 4. The scenario depicted in the figure reflects the case in which the problem solver’s knowledge state is 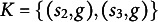 , and the problem to be solved is 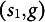 . This last does not belong to the state *K* of the problem solver. Assume that the problem is solved correctly. According to (A1) assumption, the sequences of moves will be a series of guesses and the probability of solving the problem is 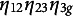 (left-hand side diagram of Figure 4). Conversely, according to (A2) assumption, only the first move will be a guess because the remaining problems 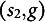 and 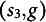 belong to the state *K* of the problem solver. Therefore, the solution path leading to the goal has a probability of 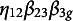 (right-hand side diagram of Figure 4).

In tasks such as the ToL test, problem solvers are required to plan in advance the solution path of each problem. If the problem solver is able to plan the solution path (i.e., the problem belongs to the state of the problem solver), in this example, MSPM1 and MSPM2 will converge on the same predictions, which means that both are able to model preplanning strategies. This is not the case if the problem does not belong to the state of the problem solver (i.e., they are not able to plan in advance the entire solution path). Unlike MSPM1, MSPM2 disentangles knowledge states on the basis of which at least some of the subproblems can be solved from knowledge states on the basis of which none of them can be solved. Therefore, if interim-planning takes place (i.e., multiple planning instances occur at various steps throughout the solution process), only MSPM2 can model it.

### 3.1 Parameter estimation

The parameters of the MSPMs can be estimated by ML via an adaptation of the expectation–maximization algorithm (Dempster et al., 1977). The details concerning the derivation of the estimation equations are provided in the Appendix section. It should be observed that (unconstrained) maximization of the model’s likelihood does not guarantee that the monotonicity conditions of the form given in (2) are satisfied. If they are violated for one or more pairs of transition probabilities 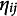 and 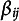 (with 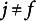 ), then the interpretation of these last parameters as “lucky guess” and “non-careless error” is not credible anymore, and the interpretation of the whole model may become problematic. Furthermore, if the model parameters are applied for carrying out adaptive assessment, convergence to the true state of the problem solver is not guaranteed when the monotonicity conditions (2) are violated (see, in this respect, Brancaccio et al., 2023). For these reasons, a constrained ML procedure was derived for both MSPM1 and MSPM2 models, where the constraints have the form of inequalities 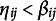 .

The unconstrained estimation of the MSPM1 was derived by Stefanutti et al. (2021) and the MATLAB code can be found at https://osf.io/qa8mg/?view_only=8b4e148300de40a6941df4a102067fc1/.

Concerning the unconstrained estimation of the MSPM2 and the constrained estimation of both the MSPM1 and MSPM2, the corresponding algorithms are available at: https://osf.io/m658x/?view_only=25c8e23729554a1a84e74ed8e50d484b.

Once estimated and validated on an appropriate sample of individuals, both the MSPM1 and MSPM2 allow for the identification of the knowledge state of an individual based on their observed response pattern. For example, the posterior probability of a knowledge state *K* given an observed jump matrix *J* can be computed using Bayes’ rule: 

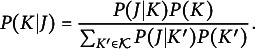

The knowledge state that maximizes 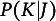 is taken as the most plausible estimate for the individual who produced the response pattern represented by *J*. This enables the assessment of individual differences. However, since 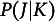 takes on different forms under assumptions (A1) and (A2), as shown by Equations (A.1) and (A.2) in the Appendix, the two models might assign different knowledge states to the same individual. Consequently, it is important to identify the best fitting model for an accurate assessment (Brancaccio et al., 2023).

## 4 Simulation study

The simulation study presented in this section has two aims. The first one is to test the parameter recovery of the MSPM2. It is worth mentioning that the parameter recovery of MSPM1 has been tested in Stefanutti et al. (2021). The second aim is to test the capability of model selection criteria to discriminate between MSPM1 and MSPM2. To pursue these aims, a simulation study is presented using the problem space of the ToL test.

### 4.1 Simulation design and data set generation

The present simulation study is based on the goal space 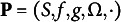 obtained from the ToL problem space. It was defined according to two criteria: (i) the goal state is problem state 11 and (ii) the solution path length ranges from 1 to 5 moves. The resulting goal space 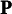 contains 20 problem states plus the goal and the failure states. Thus, 20 problems can be derived from 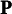 , which are all the pairs 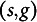 with 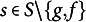 . Knowledge space 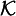 derived from 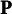 contained 1,573 knowledge states.

The data were generated under 12 different conditions displayed in Table 3. The 12 conditions result from the combination of the two assumptions (A1) and (A2) used to simulate the data, three sample sizes (300, 1,500, and 6,000), and two error levels. The three sample sizes were chosen to represent situations with small, medium, and large data sets, respectively, compared to 1,573, which is the cardinality of the structure. The two error levels were selected to represent low and medium-high error conditions.

**Table 3:** Design of the simulation study used for generating the data

Condition number	Assumption	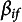	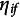	Sample size
1	(A1)	(0,0.1]	[0.9,1)	300
2	(A1)	(0,0.3]	[0.7,1)	300
3	(A1)	(0,0.1]	[0.9,1)	1,500
4	(A1)	(0,0.3]	[0.7,1)	1,500
5	(A1)	(0,0.1]	[0.9,1)	6,000
6	(A1)	(0,0.3]	[0.7,1)	6,000
7	(A2)	(0,0.1]	[0.9,1)	300
8	(A2)	(0,0.3]	[0.7,1)	300
9	(A2)	(0,0.1]	[0.9,1)	1,500
10	(A2)	(0,0.3]	[0.7,1)	1,500
11	(A2)	(0,0.1]	[0.9,1)	6,000
12	(A2)	(0,0.3]	[0.7,1)	6,000

*Note*: Column 1 displays the condition number, column 2 displays the assumption underlying the generative model, columns 3 and 4 show the error interval used for generating the data, and column 5 displays the sample size (see Section 4.1).

The error level in the data was manipulated through the 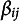 and 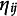 transition probabilities of the model. In particular, for each 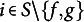 , the 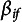 parameters were drawn at random from a uniform distribution with intervals 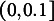 and 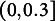 depending on the conditions. On the other hand, the 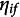 parameters were randomly drawn from a uniform distribution in the intervals 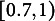 and 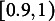 . Different intervals were chosen for the 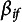 and 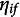 parameters because they have different interpretations. In particular, the 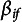 are interpreted as “careless errors” and, thus, they are expected to be small, whereas the 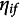 are interpreted as “non-lucky guesses” and, hence, they are expected to be close to one. The remaining 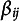 and 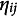 were drawn at random from a uniform distribution and then normalized to sum up to 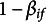 and 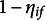 , respectively. For these reasons, conditions where 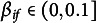 and 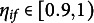 represent a low error scenario, whereas conditions where 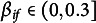 and 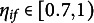 represent a medium-high error scenario. Notice that, in each scenario, each pair 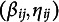 of parameters with 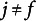 also satisfies the inequality 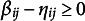 (see the Appendix).

The data were simulated under the two different assumptions (A1) and (A2). Specifically, for each subject with knowledge state 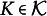 and for each problem 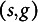 , a sequence of problem states terminating with either the failure state or the goal state is generated using the 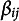 and 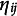 parameters. The procedure for simulating each response pattern follows the method described in Stefanutti et al. (2021, Figure 5). The only variation between (A1) and (A2) is that, in the former case, the transition probabilities used to generate the sequence are all 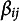 or 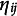 if problem 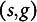 , respectively, belongs or does not belong to *K*, whereas in (A2), each transition probability is a 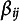 or 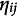 if problem 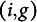 belongs or does not belong to *K*.

For each of the 12 conditions, 500 data sets were generated, obtaining a total of 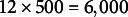 simulated data sets. Every single data set consists of a given number of response patterns, each of which is a collection of jump matrices 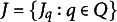 , one for each of the problems in *Q*.

### 4.2 Methods

The identifiability of the MSPM1 and the MSPM2 has been tested using an informal method consisting of the following two steps: (i) the parameters of each model were re-estimated 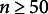 times on the same data set, each time starting from a different initial set of parameters values, until 50 different estimations with equal likelihood were obtained and (ii) the standard deviations of the parameter estimates obtained in the 50 estimations were computed. It is expected that the standard deviation of the estimates is zero up to round-off when parameters are identifiable. Typically, this procedure is applied in those situations in which a formal test of (local) identifiability is not available (see, e.g., Stefanutti et al., 2020, 2021).

In each of the 12 conditions, 500 data sets were simulated and both the MSPM1 and MSPM2 were fitted to the data. The goodness of the parameter recovery of the MSPM2 was tested in those conditions in which (A2) was the assumption under which the data sets were generated. In particular, the bias and the variance of the parameter estimates across the 500 simulated data sets were computed. The estimated bias for each 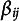 was computed as follows: 
(3)

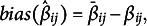

where 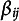 is the parameter value used for generating the data and 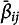 is the arithmetic mean of the 500 estimates of the 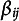 parameters. The standard deviation of the estimate 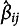 was obtained as follows: 
(4)

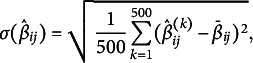

where 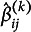 is the estimated value of the transition probability for the *k*-th simulated sample. It is worth noticing that 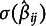 is also the standard deviation around 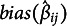 . The bias and variance for 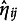 were computed in the same way.

Moreover, as an index of the expected amount of information in the data to estimate the 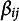 parameters, the “true” marginal problem probability was computed. Let 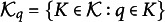 be the collection of all the knowledge states containing problem *q*. Then, for each 
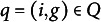
 , the “true” marginal problem probability 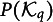 is given by 

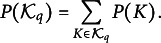



Similarly, the expected amount of information in the data to estimate the 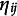 parameters is given by the probability 

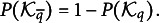

For simplicity, both 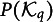 and 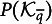 are called “true” marginal probability of the problem all along the manuscript, because it is the probability of the states used to generate the data sets.

To test the capability of the model selection criteria to identify the assumption under which the data were generated, the Akaike information criteria (AIC; Akaike, 1973) computed for the two estimated models were compared to one another. The proportion *p* of times over the 500 replications in which the AIC selects the model that is consistent with the generative assumption was computed in all the conditions. For instance, a 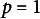 in Condition 1 means that the AIC selects the MSPM1 for all the 500 data sets, whereas a 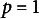 in Condition 7 means that the MSPM2 is selected for all the 500 data sets.

### 4.3 Results

In the MSPM1, the two parameters 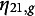 and 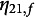 may have identifiability problems, because an application of the two-step method illustrated at the beginning of Section 4.2 resulted in a standard deviation of 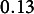 . Concerning the MSPM2, no identifiability issues have been observed.

The following describes the parameter recovery of the MSPM2, which was estimated on the data sets simulated under assumption (A2). For the parameter recovery of the MSPM1, the reader can refer to Stefanutti et al. (2021).

**Figure 5:**
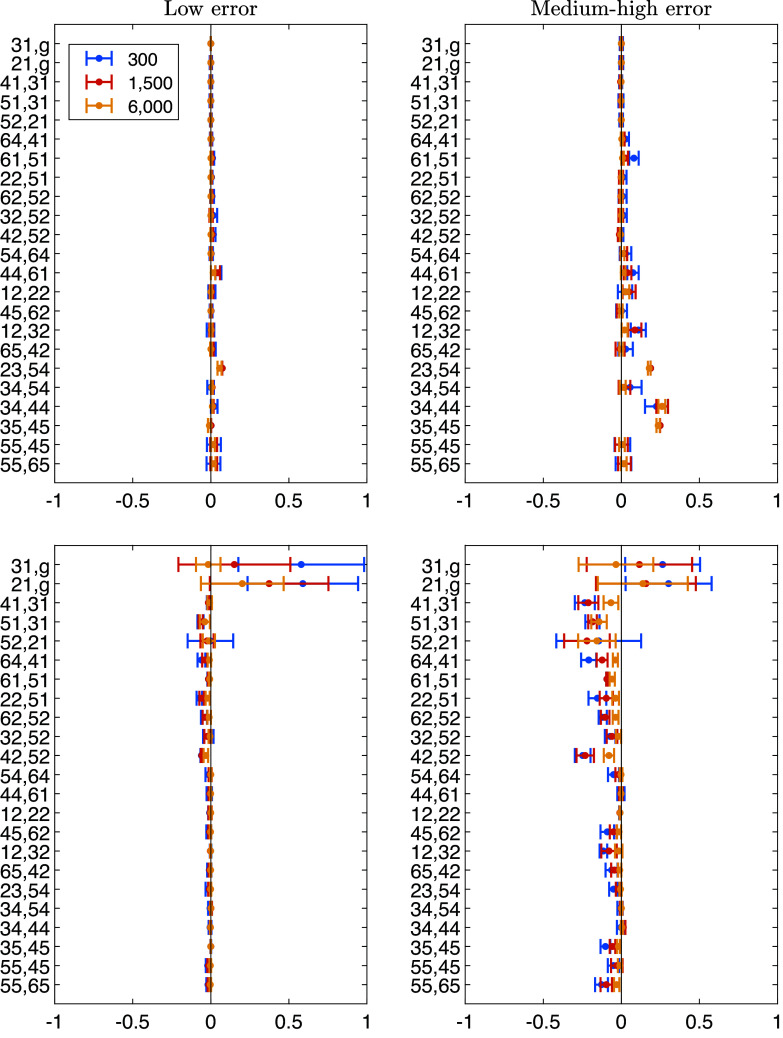
Parameter recovery of the MSPM2’s 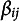 (top panels) and 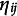 parameters (bottom panels) across three sample sizes: 300 (blue), 1,500 (red), and 6,000 (yellow). *Note*: In each panel, the pairs 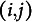 representing the parameter estimates 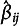 or 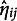 , with 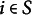 and 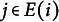 , are along the vertical axis, while the bias of the parameter estimates is measured along the horizontal axis. The left and right panels represent low and medium-high error conditions, respectively.

Figure 5 displays the bias of the MSPM2 parameter estimates. The top panels correspond to the 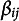 transition probabilities, while the bottom panels correspond to the 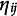 transition probabilities. The left and right panels represent low and medium-high error conditions, respectively. In each panel, the pairs 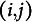 representing the parameter estimates 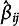 or 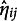 , with 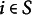 and 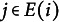 , are along the vertical axis, while the bias of the parameter estimates is measured along the horizontal axis. For convenience, only the free transition probabilities are shown (e.g., if the constraint 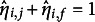 holds then only 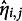 is displayed). In the condition with 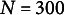 and low error (Condition 7), the bias appears to be negligible for all parameters 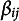 and most parameters 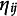 . The only exceptions are parameters 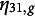 and 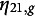 , which exhibit biases as large as 0.58 and 0.59. These biases might stem from insufficient information in the data, as explained below.

**Figure 6:**
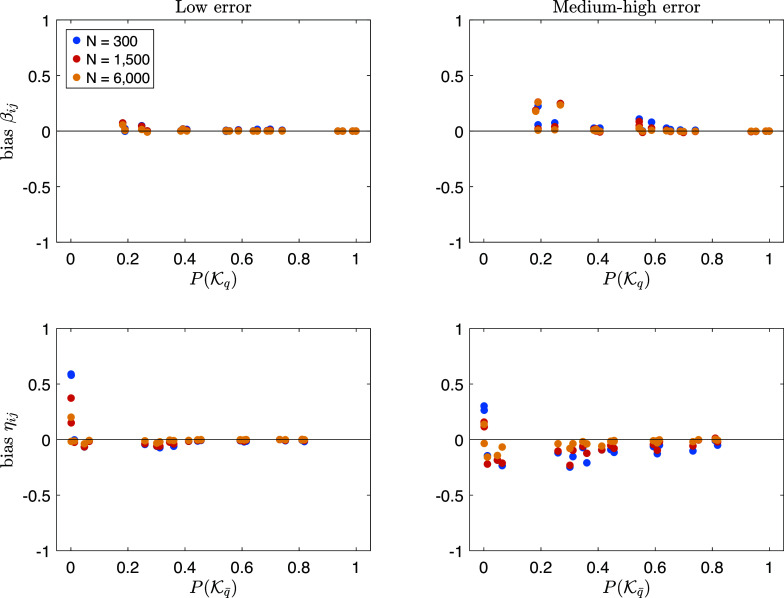
Bias of 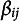 (top panels) and 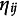 (bottom panels) as a function of their marginal problem probability (*x*-axis), across three sample sizes: 300 (blue), 1,500 (red), and 6,000 (yellow). *Note*: The left panels correspond to low error conditions, while the right panels correspond to medium-high error conditions.

Figure 6 displays the bias of the parameter estimates (vertical axis) as a function of the “true” marginal problem probability (horizontal axis). As in Figure 5, the top and bottom panels correspond to parameters 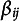 and 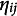 , respectively, while the left and right panels represent low and medium-high error conditions. The transitions whose parameters have the largest bias are exactly those with the lowest marginal probabilities of being observed. For instance, 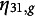 and 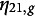 have marginal problem probabilities as low as 0.0012 and 0.0007, respectively (bottom left panel of Figure 6). In other words, the probabilities to simulate a knowledge state 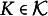 that does not include problems 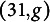 or 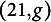 are 0.0012 and 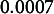 , respectively. As a result, in a data set with a sample size of 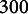 , the expected frequencies for these transitions are 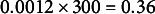 and 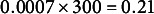 , corresponding to less than one individual per transition.

As the sample size increases, the number of individuals contributing to the estimation of the parameters increases and the bias of the parameter estimates decreases (red and yellow data points in Figure 5). Two aspects are worth noting. First, even with samples sizes of 1,500 and 6,000, the number of individuals available for estimating parameters 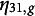 and 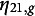 remains rather small (i.e., 1.8 and 7.2 for 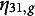 ; 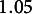 and 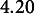 for 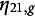 ). Second, these two parameters correspond to transitions that are one step away from the goal state. In the context of the ToL task, these transitions are almost never failed in the general population. Specifically, the probability that an individual fails a one-move problem is close to zero, making such transitions highly infrequent.

Concerning the medium-high error conditions, with the sample size held fixed, the biases of the parameter estimates increase. Interestingly, however, the bias is slightly higher across all transitions, even though the maximum bias is smaller compared to the condition with a smaller sample size.

Concerning conditions from 7 to 12, Table 4 displays the mean absolute bias for 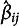 and 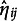 calculated across all transitions (second and third columns), and their mean standard deviation (fourth and fifth columns). As expected, the biases are smaller in the low error conditions (i.e., 7, 9, and 11), rather than the high error conditions (i.e., 8, 10, and 12). It is also worth mentioning that in all conditions 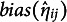 is bigger than the 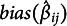 . The last result can be explained by looking at Figure 6, where it is clear that there is more information for estimating the 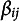 parameters than for estimating the 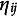 parameters. Indeed, even for the 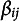 parameters with the least information, there is a marginal problem probability of about 0.20, whereas for the 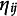 parameters with the least information, there is a marginal problem probability of about zero.

**Table 4:** Bias obtained in the simulation study for MSPM2 under each condition of sample size and error level

Condition	Mean 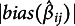	Mean 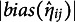	Mean 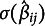	Mean 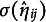
7	0.012	0.072	0.015	0.051
8	0.050	0.117	0.030	0.064
9	0.009	0.041	0.007	0.042
10	0.044	0.088	0.019	0.059
11	0.006	0.017	0.005	0.024
12	0.035	0.041	0.010	0.043

*Note*: Columns 2 and 3 display the mean absolute bias for 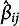 and 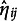 , respectively, while columns 4 and 5 show the mean values of the corresponding standard deviations.

Finally, in all conditions, the proportion *p* of instances in which the AIC selects the model consistent with the assumption that generated the data is 1, with the sole exception of Condition 8, where 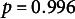 . It is worth noting that, since MSPM1 and MSPM2 have the same number of parameters, comparing their AIC values is equivalent to directly comparing their likelihoods.

### 4.4 Discussion

Overall, MSPM2 demonstrates good parameter recovery performance, particularly for the majority of transitions. Even under relatively small sample sizes (i.e., 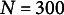 ), estimates for both 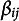 and 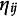 parameters are generally unbiased, with deviations emerging only in those transitions where failure is intrinsically rare (i.e., with very low variability in the data). Importantly, the two parameters susceptible to bias—namely, 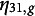 and 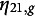 —refer to transitions toward the goal state in single-move problems, which are of little behavioral importance as these transitions are very rarely failed in the general population. As such, these aspects do not compromise the interpretability or practical utility of the model.

A potential limitation of this simulation study should also be mentioned. Indeed, data sets were obtained by using only 12 generating parameter sets. While this ensures internal consistency, it could not adequately capture the full variability of parameters encountered in real-world contexts. Future work could address this by employing a broader and more systematically varied set of generating conditions to enhance generalizability.

## 5 Empirical application

The literature on the development of planning ability highlights that there are some transition ages in which the performance at tower tasks and the type of planning employed may change. Specifically, children aged 4–8 seem to have lower performance than older children (McCormack & Atance, 2011), with some authors suggesting that 4-year-old children have qualitatively different response processes (Kaller et al., 2008; McCormack & Atance, 2011). Whereas Asato et al. (2006) report that from 8 years and older, the pre-planning time starts to increase, reaching adult-level performance around 14–15 years old (Luna et al., 2004).

In the previous section, a suggestion was made that the assumption behind MSPM1 represents a response behavior in which the planning process occurs predominantly at the onset of the solution process. Conversely, the assumption behind MSPM2 would represent a response behavior where multiple planning instances occur at various steps throughout the solution process.

Merging the insights gained from the literature on the development of planning ability with those of MSP models, the following three hypotheses seem plausible:

1. Children younger than 8 years old do not preplan their responses but they might resort to an interim planning. Therefore, the MSPM2 should better fit the data than MSPM1.
2. Children aged 9–13 begin to preplan their moves, although they may still demonstrate inconsistencies in their strategy. Therefore, both MSPM1 and MSPM2 are expected to fit the data.
3. Individuals older than 14 years old should consistently preplan their responses, therefore, MSPM1 should better fit the data than MSPM2.

The analysis of the response times should also bring evidence in favor of these hypotheses. In fact, the preplanning time is expected to be longer when the problem solution is planned in advance (i.e., before moving) and it is expected to be shorter when the problem is solved with an interim-planning strategy (i.e., between one move and the other). A response time analysis can serve as an external criterion for assessing the validity of the models.

### 5.1 The assessment tool: Adap-ToL

This study is part of a research project founded by the Italian Ministry of Education and Research aimed at the development of an intelligent system, named PsycAssist (de Chiusole et al., 2024), for the adaptive assessment of executive functions and fluid intelligence among general and clinical populations. The study presented here shows the results obtained from the application of the MSPM1 and MSPM2 to the data collected with one of the tools available on PsycAssist, which is Adap-ToL.

Adap-ToL is a test for the assessment of planning skills in individuals aged four and above. It resembles the ToL test, where participants move beads from an initial setup to a goal configuration, within a set number of moves and following specific rules. Unlike the traditional ToL test, where the initial state is constant among the problems but the goal state varies, Adap-ToL maintains a fixed goal state while varying the initial configuration. This was designed to maintain coherence with the PKST definition of a problem in a goal space.

Different versions of the test were designed. Among all the 35 problems having the same tower configuration (i.e., configurations in the first row of Figure 1) as the goal state, those with a number of moves 1–5 were used for creating a version of the test designed for individuals aged 4–8; those with a number of moves 1–6 were used for creating a version of the test designed for individuals aged 9–13; and all the 35 problems were used in the version for individuals older than 14.

**Figure 7:**
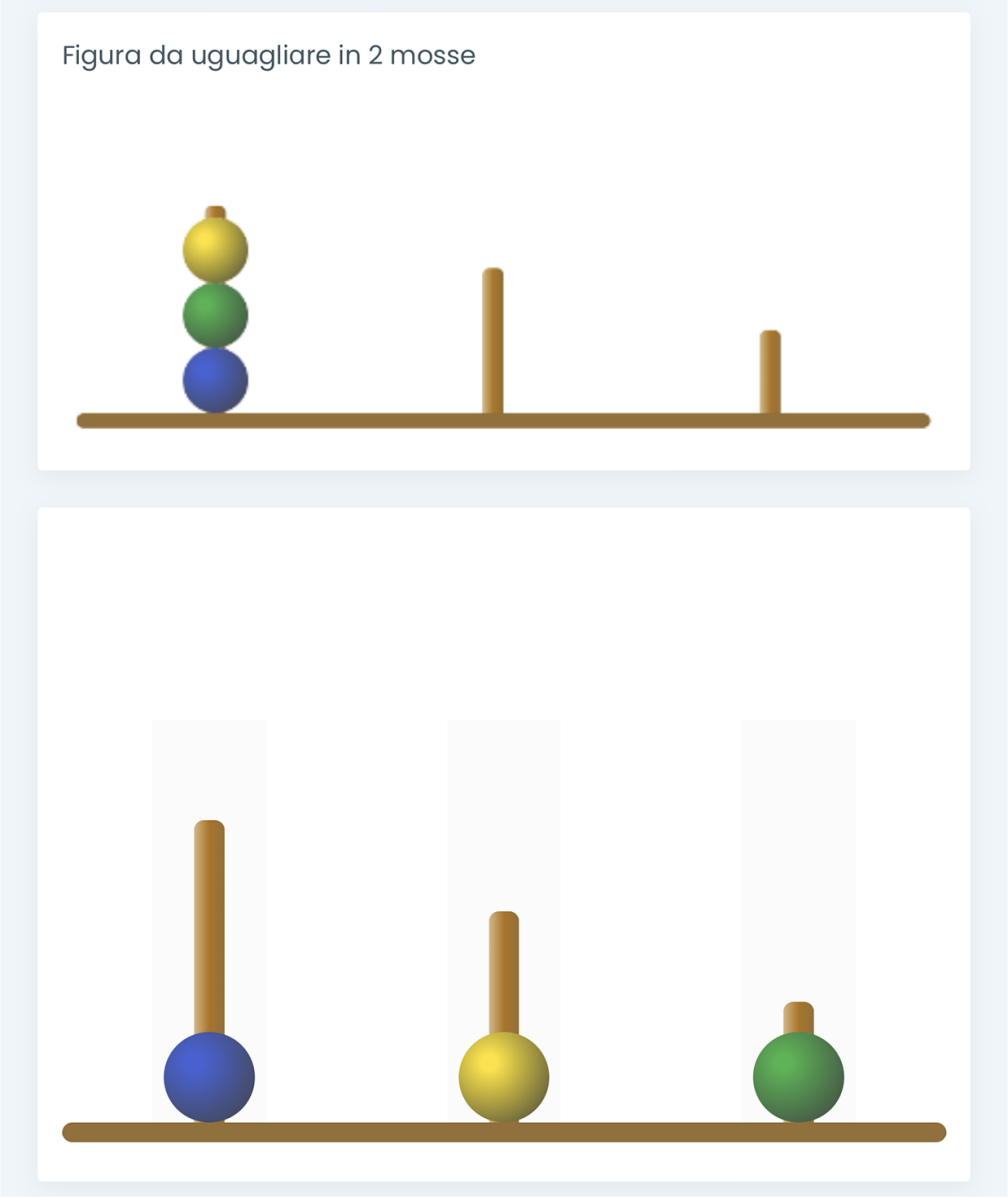
One of the problems in Adap-Tol. *Note*: Each problem of the test consists of two images, one above the other. The upper image represents the configuration of the goal state, whereas the lower image represents the initial configuration of the problem. The task is to replicate the goal state from the initial configuration, within a specified minimum number of moves (specified at the top of the upper image). In the depicted configuration, “Figura da uguagliare in 2 mosse” translates to “Figure to be matched in 2 moves”). Acting on the lower image, participants tap the screen twice to make a move: first to select the bead, and then to choose the peg in which to move the bead.

The test is administered by using a tablet (IoS operating system with a 10.9-inch screen) oriented vertically. Each problem of the test consists of two images, one above the other (Figure 7). The upper image represents the configuration of the goal state, whereas the lower image represents the configuration of the initial state of the problem. The task is to replicate the goal state from the initial configuration, within a specified minimum number of moves (specified at the top of the upper image). Acting on the lower image, participants tap the screen twice to make a move: first to select the bead, and then to choose the peg in which to move the bead. The problem is successfully solved when the goal state is reached within the minimum number of moves.

The test is introduced by a brief animated video (95 seconds), which presented the test, explained the task, and gives the following two rules: (a) the highest peg can hold three beads, the central peg can hold a maximum of two beads, and the shortest peg can hold only one bead and (b) a bead cannot be moved if it has another bead on its top. The system gives to the test-taker a feedback in case of the violation of one rule, an excessive number of moves made to complete the task, and when the task is correctly executed. Each feedback is presented within a colored box (red, orange, or green, respectively) by using a simple text and an emoticon. For children who cannot read yet or who may face challenges in reading, the evaluator reads the message aloud.

After the video presentation and before the test administration, the test-takers were presented with four practice problems. If the test-taker struggled to find any answer to a problem, the evaluator could decide to skip the problem and move to a next one. It is worth noticing that the decision to skip a problem was made solely by the evaluator and only when the test-taker was unable to reach the goal configuration and showed signs of frustration. The problems were presented one at a time in random order.

As in traditional ToL tests, the “pre-planning time” (i.e., the time elapsed between the presentation of the problem and the first click), the “execution time” (i.e., the time elapsed between the first click and the completion of the problem), the “total time” (i.e., the sum of the pre-planning and execution times), and the accuracy of each problem were recorded. Concerning accuracy, the problem is considered correctly solved when the goal state is reached within the minimum number of moves; otherwise, it is wrong.

Different from other ToL tests, where at maximum three attempts are given to solve a problem, Adap-ToL prescribes only one attempt. Moreover, Adap-Tol registers the whole solution path made by a problem-solver. This aspect is fundamental for applying MSPM1 and MSPM2.

**Figure 8:**
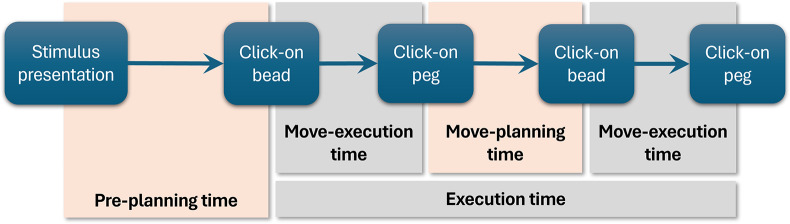
Decomposition of the solution process of a 2-move problem in the “traditional” pre-planning and execution times and the new move-execution and move-planning times.

The computerized version of the test allows for registering the time at each click made by a test-taker. Consequently, other two types of sub-times can be registered, that is: (i) the “move-execution time,” which is the time spent moving a bead, is computed as the time elapsed from the click on a bead to the click on a peg and (ii) the “move-planning time,” which is time spent selecting a new bead to move, is computed as the time from the click on a peg to the click on a new bead. Figure 8 shows how the entire solution process of a 2-move problem is decomposed among the “traditional” pre-planning and execution times and the new move-execution and move-planning times.

### 5.2 Participants

Adap-Tol was administered to a sample, extracted from the general population, of 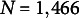 participants (Female 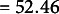 %, Age range: 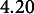 – 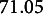 years, Mean age: 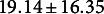 years). Data were collected in different regions of the North, Centre, and South of Italy (i.e., Abruzzo, Basilicata, Calabria, Emilia-Romagna, Lazio, Liguria, Lombardia, Marche, Puglia, Toscana, Umbria, and Veneto) and schools of all grades as well as adults in the general population were involved. Before participating in the study, participants and/or their parents received detailed information about the study purposes and procedures, and they (or their parents/legal guardians) provided a signed informed consent, in accordance with the Declaration of Helsinki.

Data from 69 people with a diagnosis (e.g., ADHD, autism spectrum disorder, and intellectual disability) were removed. The removal of individuals with a diagnosis is due to the fact that this study focuses on assessing the sensitivity of the two models to age-related differences in the general population. In fact, including individuals with specific difficulties could bias the results, potentially making older participants perform more similarly to younger ones. No problems were skipped, thus the data set does not have any missing answers. Moreover, a maximum age limit was set at age 57. Thus, data from 105 participants aged 57 and above were excluded. This age limit was chosen to (i) prevent performance decline due to age-related factors and (ii) ensure a sample size comparable to the other age groups. The final sample was composed of 1,053 respondents (Female 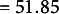 %, Age range: 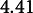 – 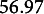 years, Mean age: 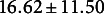 years). Age group 4 to 8 was composed of 269 children (Female = 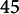 %, Mean age: 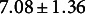 ), age group 9 to 13 was composed of 343 children (Female = 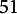 %, Mean age: 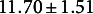 ), age group 14 to 57 was composed of 441 individuals (Female = 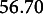 %, Mean age: 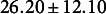 ).

A two-proportion *z*-test was used to investigate any significant difference in the proportion of correct responses between female and male children in each age group (Type I error probability 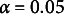 ). The proportion of correct responses was computed considering the 20 problems that were presented to all respondents regardless of their age. The Benjamini–Hockberg (Benjamini & Hochberg, 1995) correction for multiple comparison was applied. The results are reported in Table 5. After correcting the *p*-values for multiple comparisons, the difference in the proportion of correct responses in female and male participants remained significant in the 4–8 group and in the 30–57 group. In the former case, female participants performed significantly better than male participants, while the opposite effect was observed in the latter case.

**Table 5:** Proportion of correct responses of female (F) and male (M) participants in each age group

Age group	F ( 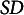 )	M ( 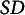 )	*p*-value
4–8	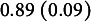	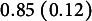	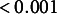
9–13	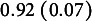	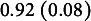	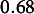
14–29	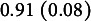	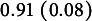	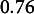
30–57	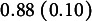	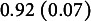	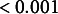

*Note*: The *p*-values reported in the last column are already corrected according to the Benjamini–Hockberg correction.

### 5.3 Data analysis

For maximizing the number of problems shared by the three age groups, only problems with 1–5 moves were considered.

Both MSPM1 and MSPM2 were fitted to the data of each age group by using the knowledge space 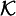 and goal space 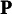 of the ToL introduced in Section 4.

Hypotheses (H1)–(H3) were tested by comparing both the absolute and the relative goodness-of-fit of the models. The absolute goodness-of-fit of the models was tested by the Pearson Chi-square statistic. In particular, a parametric bootstrap procedure (Efron, 1979) over 500 replications was used to compute the *p*-value of the Chi-square. When both models fitted the data, the AIC index and Bayes Factor were used to select the best model. The Bayes factor was approximated using the Bayesian information criterion (BIC; Schwarz, 1978).

Concerning the response time analysis, the entire time spent to complete a problem was split into three sub-times. The first one is the “pre-planning time,” which is the time spent from the presentation of the task to the first click on a bead. The second one is the “move-execution time,” which is the time spent from the click on the bead to the click on the peg. The third one is the “move-planning time,” which is the time spent from the click on a peg to the click on a new bead. It follows that the sum of these three times equals the total time spent to complete the problem. Thus, for each participant, a standardized version of the three sub-times can be obtained by dividing each of them by the total time. Only the response times of problems correctly solved were considered in this analysis. Moreover, average values across test-takers were obtained separately for each age group.

Standardizing sub-times in this way allows for obtaining indexes whose range is in the interval 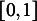 , and that express the average proportion of time spent on the specific “action.” Since the total time spent to solve a problem strictly depends on the number of moves, these indexes might be misleading if used for comparing the response behavior of the same group (as well as of different groups) to different problems. Nonetheless, these indexes should provide useful insights when used for comparing the response behavior of different age groups while keeping the problem fixed.

### 5.4 Results

The MSPM1 obtained a strong rejection (bootstrapped *p*-value 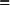 0.004), whereas the MSPM2 adequately fit the data with a bootstrapped *p*-value 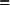 0.312 on the group aged 4–8.

Concerning age group 9–13, a first attempt to fit the models to the data obtained a strong rejection for both models, with bootstrapped *p*-values 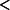 0.005. To investigate the lack of fit of the models, the standardized residual associated with the Chi-squared statistics was computed. In both the MSPM1 and MSPM2, the same response pattern had the largest standardized residual (i.e., 10.0 for the MSPM1 and 10.6 for the MSPM2). Extreme outliers can distort the overall model fit, especially when using goodness-of-fit measures like Chi-square. Such a response pattern can be considered as an extreme outlier. Indeed, an additional qualitative check of the response pattern revealed frequent violations of the model’s expected difficulty hierarchy: easier problems (requiring 1–3 moves) were failed, while a more complex seven-move problem was solved. In addition, most failures occurred at the final step before reaching the goal. Therefore, it was excluded from the analysis. This decision follows standard practices to ensure a reliable evaluation of the model parameters (Rousseeuw & Leroy, 1987).

After removing the response pattern, both MSPM1 and MSPM2 exhibit a good fit to the data, with bootstrapped *p*-values of 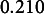 and 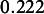 , respectively. Concerning the model comparison, the AIC index of MSPM1 is 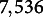 , while that of MSPM2 is 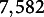 . Thus, MSPM1 is the selected model for individuals aged 9–13. The Bayes factor also shows evidence in favor of MSPM1 with a value of 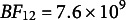 .

Finally, in the age group 14–57, both MSPM1 and MSPM2 fit the data well with bootstrapped *p*-values of 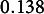 and 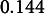 , respectively. The AIC comparison selects MSPM1 with an AIC statistic of 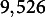 , compared to 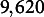 for MSPM2. The Bayes factor shows evidence in favor of MSPM1 with a value of 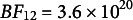 .

**Figure 9:**
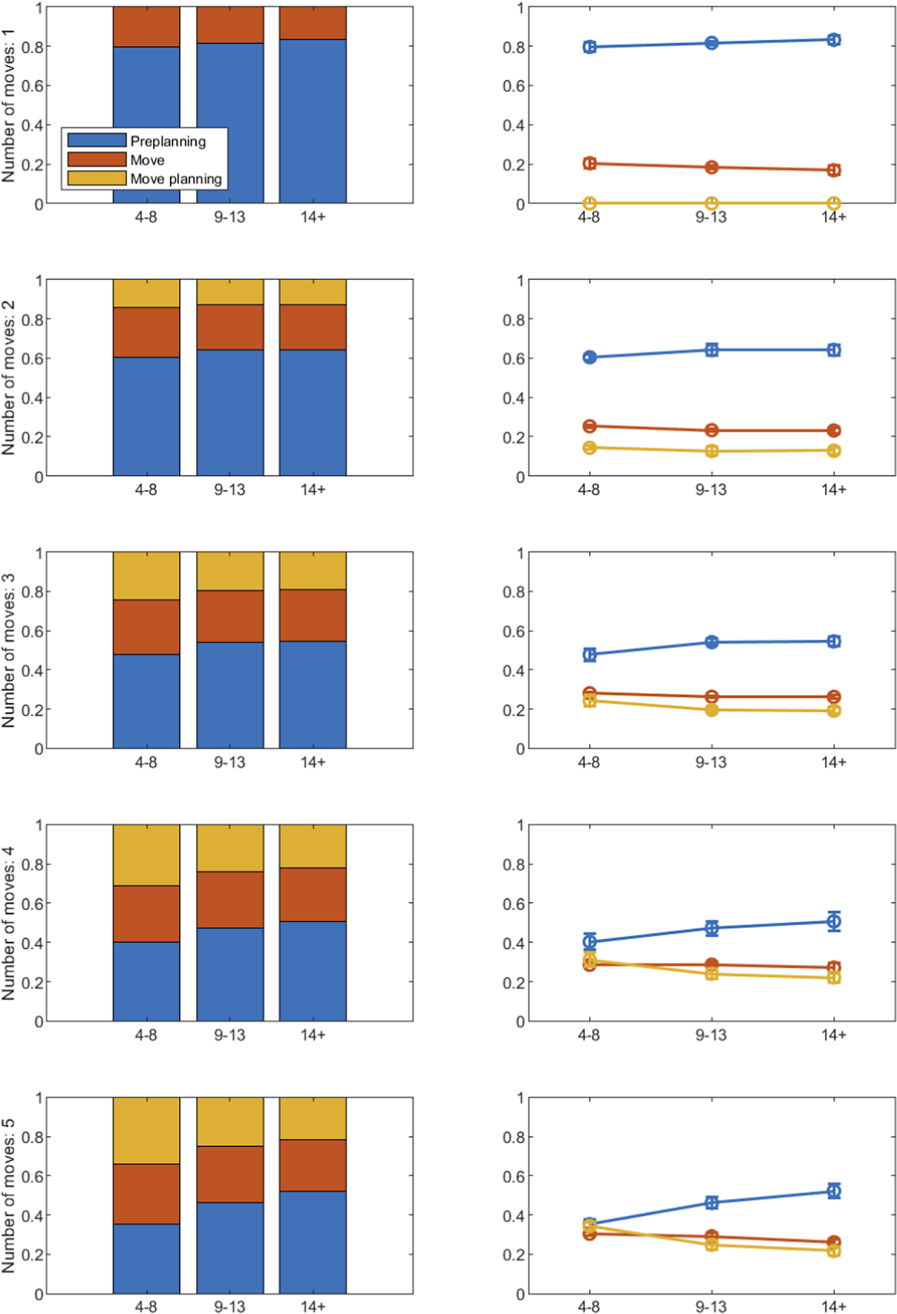
Results on the response time descriptive analysis. *Note*: See text for more details.

Figure 9 shows the results of the response time descriptive analysis obtained with the three age groups (*x*-axis in each panel). The *y*-axis of each panel gives the proportion of the total time spent to complete a problem. Rows display the results obtained with problems having a different number of moves, going from 1 (top panel) to 5 (bottom panel). Pre-planning time is in blue, move-execution time in yellow, and move-planning time in red. The three standardized sub-times are represented by stacked bar graphs (panels at the left) and line plots (panels at the right) with error bars representing standard deviations. Stacked bar graphs can be useful for analyzing how participants of the three age groups distributed the response time among the three actions. Whereas line plots can serve for studying the trend of each action across age groups.

In problems with a smaller number of moves (i.e., 1 and 2), a similar response time behavior was observed among the three age groups. In particular, most of the total time is spent on pre-planning. It is worth noticing that in 1-move problems the proportion of the total time spent in pre-planning time and that spent in move planning coincide (this is why the last does not appear in the graph). Concerning the trend across age groups, a slightly monotonic increase was observed for the proportion of the total time spent in pre-planning time, even if with a small amount (the gain is 0.04 in both 1-move and 2-move problems).

Instead, the response time behavior seems different across age groups when the number of moves increases. In 5-move problems, despite the proportion of time spent is executing the move is quite similar in the three groups (the biggest drop is 0.02), the proportion of time spent on pre-planning increases (with a gain of 0.17) and the proportion of time spent in move-planning decreases (with a drop of 0.13) as age increases. Moreover, it seems that children aged 4–8 spent a proportion of time in pre-planning and in move-planning that is very similar (i.e., 0.35 and 0.34, respectively). Conversely, individuals older than 14 spent a proportion of time in pre-planning and in move-planning, which is quite different, with the former greater than the latter (i.e., 0.52 and 0.21, respectively). In summary, the outcomes derived from both the response time and solution process analysis support Hypotheses (H1)–(H3).

### 5.5 Discussion

MSPM1 and MSPM2 are based on different assumptions about how planning occurs during problem solving. MSPM1 assumes that planning takes place predominantly at the onset of the solution process, leading to a more rigid execution of an initial strategy. In contrast, MSPM2 allows for multiple instances of planning throughout the task, supporting a more flexible and adaptive approach.

The superior fit of MSPM2 in the 4–8 age group suggests that younger children do not rely solely on an initial plan but rather adjust their strategy dynamically as they progress. In contrast to this age group, the results for older participants indicate a shift in planning behavior. For individuals aged 9–13, both MSPM1 and MSPM2 exhibit a good fit to the data, but model comparison criteria favor MSPM1. The lower AIC value and the strong Bayes factor in favor of MSPM1 suggest that, in this age range, planning tends to occur primarily at the onset of the solution process, with less reliance on dynamic adjustments during task execution. A similar pattern emerges in the 14–57 age group, where MSPM1 remains the preferred model based on AIC and Bayes factor comparisons.

These findings align with the idea that, as individuals develop greater cognitive control and experience with problem solving, they rely more on pre-planned strategies and execute solutions with reduced need for mid-task adjustments. This developmental trend supports the interpretation that younger children engage in more iterative and corrective planning, whereas older individuals increasingly favor an upfront, goal-directed approach to problem solving.

## 6 General discussion

Beyond providing the algorithms for the ML estimation of the model based on the interim-planning, this manuscript brings evidence in favor of the usefulness of considering the sequences of moves undertaken by the respondents in solving tower tasks.

Considering only the accuracy performance at tower tasks might be reductive and might not allow for getting a comprehensive understanding of the cognitive processes involved in the completion of the task. By capitalizing the information provided by the sequences of moves, the approach presented in this contribution allowed for the distinction between respondents of different ages who employ different strategies for solving tower tasks.

The results of the empirical application showed that the model based on the interim-planning assumption best fitted with respondents aged 4–8, while that based on the pre-planning assumption best fitted with respondents aged 14 on. These results are consistent with cognitive theories asserting that young children have not yet entirely developed the executive functions related to planning (Asato et al., 2006; Kaller et al., 2008; Luna et al., 2004; McCormack & Atance, 2011).

Interestingly, both models are plausible given the data of respondents aged 9–13. This result might be explained by considering both the intra- and inter-individual differences. From the intra-individual perspective, the same individual might change their solution strategy depending on the difficulty of the problem. For instance, they might use a pre-planning strategy for problems with a lower number of moves, while they might use an interim-planning strategy when facing problems with a higher number of moves. From the inter-individual perspective, the respondents in this age group might differ from one another with respect to their solution strategy, presenting a high degree of heterogeneity. Future studies employing a think-aloud protocol might provide further evidence about the planning strategies used by the individuals in this age group. In this light, a new model able to combine the pre-planning and interim-planning assumptions would be useful to better understand the potential occurrence of the shifting between planning strategies. Another possibility to extend the models considered so far would be to consider as knowledge states subsets of solution paths rather than subsets of problems.

This manuscript demonstrated the usefulness of the two MSPMs for both the accuracy and the observed sequences of moves in the discrete time. The preliminary results on time performances suggest that a promising future direction could be the development of Markov models in the continuous rather than discrete time, in analogy to Anselmi et al. (2021).

In summary, the models based on the pre-planning and interim-planning assumptions are able to discriminate between respondents of different ages and provide insights into the cognitive processes involved in the solving of tower tasks.

## Footnotes

1

 is closed under union if for every subset  , the union of all the elements in  is still in  .

## Appendices

### A MSPM parameter estimation

The parameters of both MSPM1 and MSPM2 models can be estimated by ML via an adaptation of the expectation–maximization (EM) algorithm (Dempster et al., 1977). The observed data set is a collection  of *response patterns*, each of which is a collection  ,  of jump matrices  obtained from the observed responses of problem solver *v*. The collection contains one jump matrix for each problem in  .

The “complete” (though partially observable) data set is a collection of pairs  , where  and  are, respectively, the observed response pattern and the latent knowledge state of problem solver *v*. Let  denote the vector of the model parameter estimates obtained at step  of the EM algorithm (such vector comprises the estimated values of all the  and  parameters, as well as the probabilities of the knowledge states). In each step *m*, the EM algorithm improves the estimate  by maximizing an expected value of the so-called “complete (log)likelihood” of the model. It is convenient to have available the following definitions. For  define the matrix sums

Moreover, for  , let

Finally, for  define the two collections:

and  . For both MSPM1 and MSPM2, the complete log-likelihood has form

where the explicit form of  depends on which of the two simplifying assumptions (A1) or (A2) is applied to the transition probabilities. As shown in Stefanutti et al. (2021), it follows from the Markov property, and from (A1) that (let  denote the complement  )

Therefore, under the simplifying assumption (A1), one obtains the model:

(A.1)

Concerning (A2), for  , define the collection

Then, from the Markov property and the simplifying assumption (A2), it follows that

Therefore, under assumption (A2), the following model is obtained:

(A.2)

In both models, the general equation of the conditional expectation of *L*, given the observed data set  and the parameter estimates  , is

where

is the posterior probability of  given response pattern  and the parameter estimate  obtained at step *m* by an application of Bayes theorem, and  is obtained by an application of Equation (A.1) to  .

The conditional expectation  can conveniently be decomposed into two terms  and  such that  :

and

Then, the parameter vector  maximizes  if and only if it maximizes both  and  . Each of the two terms is maximized by setting to zero its first partial derivatives with respect to the model’s parameters. In this respect, we observe that  only depends on the  knowledge state probabilities, whereas  only depends on the  and  parameters (through the probability  ).

Concerning  , its maximization is constrained by the equality  Then, by using Lagrange multipliers for this constraint, the quantity that is actually maximized is

where  is the Lagrange multiplier for the equality constraint at hand. Differentiating with respect to  , setting to zero, and solving for  one obtains the equation

which is the improvement of the ML estimate of  at iteration  of the EM algorithm.

Turning to the  term, its maximization is constrained too, because the two equalities

must stay true for all  . Thus, using again Lagrange multipliers, the quantity that is actually maximized is

where  is the Lagrange multiplier for the equality constraint on the  parameters, whereas  is the Lagrange multiplier for the equality constraint on the  parameters. Then, for  , the derivative of  with respect to the  parameter turns out to be

Similarly,

#### A.1 MSPM2 parameter estimation

Let  be defined as in Equation (A.2) (Assumption (A2)). Differentiating  with respect to  gives

Differentiating  with respect to  gives

Therefore, after some algebra, we obtain the first partial derivatives of  with respect to  and

and

Equating both derivatives to zero and solving for  and  , respectively, one has

(A.3)

and

(A.4)

Summing over all  in both equations, we obtain that

and

Plugging the right-hand side of the first (respectively, last) of these two equations into Equation (A.3) (resp., Equation (A.4)), one gets

and

which are the equations for the improvement of the ML estimates of the two parameters  and  in the MSPM2 model.

### B Estimation with monotonicity constraints

In both MSPM models, the values of parameters  and  are interpretable only if the monotonicity constraint

(B.1)

holds true for each  and each  . In this section, a method is presented for achieving a constrained ML estimation of the parameters of MSPM1 and MSPM2. The method is an application of a modified EM procedure, in which the constraints of the form (B.1) are satisfied in each step of the iterative algorithm. It is assumed that initial values  of the parameters are chosen so that the constraint (B.1) is satisfied for all pairs  .

In each of the two models MSPM1 and MSPM2, and in each step *m* of the EM procedure, new “tentative estimates”  and  are obtained by an application of the updating equations for unconstrained estimation of the model’s parameters (see previous sections). If the inequality  holds true, then one simply sets  and  and a new iteration of the EM takes place (unless the termination criterion is reached). If, instead the previous inequality is false, then, under the constraint in (B.1), the only allowed alternative is  . This last situation casts down to a problem where only equality constraints are imposed. Using yet again Lagrange multipliers, the quantity that is actually maximized in this case is

where each  is the Lagrange multiplier for the equality constraint on the  parameters, each  is the Lagrange multiplier for the equality constraint on the  parameters, and each  is a Langrange mutiplier related to equality constraints of the form  .

The Lagrangian  is maximized by setting to zero its first partial derivatives with respect to the parameters  and  . For  , the derivative of  with respect to the  parameter turns out to be

Similarly,

#### B.1 Constrained estimation for MSPM1

Under assumption (A1), one obtains the closed forms

(B.2)

and

(B.3)

By setting to zero the right-hand terms of both equations (B.2) and (B.3), and solving each of them for  , we obtain:

Since we are considering the case  , by equating the two parameters, from the previous equation, we obtain

Recalling that  , and solving for  ,

(B.4)

Summing across all  ,

Since  , solving for  , gives

By plugging the right-hand term of this last equation into (B.5), we obtain the updating equation for  (and  ):

#### B.2 MSPM2 constraint estimation

Under (A2), one obtains

and

By setting to zero the right-hand terms of both equations, and solving each of them for  , we obtain:

Since, again, we are considering the case  , by equating the two parameters, from the previous equation, we obtain

Solving for

(B.5)

Summing over all  ,

Since  , solving for  ,

We thus obtain that
