# Two Markov Solution Process Models for the Assessment of Planning in Problem Solving—ERRATUM

**DOI:** 10.1017/psy.2025.10070

**Published:** 2026-03-27

**Authors:** Andrea Brancaccio, Debora de Chiusole, Ottavia M. Epifania, Pasquale Anselmi, Matilde Spinoso, Noemi Mazzoni, Alice Bacherini, Matteo Orsoni, Sara Giovagnoli, Irene Pierluigi, Mariagrazia Benassi, Giulia Balboni, Luca Stefanutti

It has been drawn to the publishers’ attention that the author declarations were missing from the published paper. The declarations are as follows:

## Data Availability

The datasets analyzed in the current study are available from the corresponding author upon reasonable request.
